# Screening and surveillance recommendations for central nervous system hemangioblastomas in pediatric patients with Von Hippel-Lindau disease

**DOI:** 10.1007/s11060-024-04676-5

**Published:** 2024-04-22

**Authors:** Anna Laura Knoblauch, B.-I. Blaß, C. Steiert, N. Neidert, A. Puzik, E. Neumann-Haefelin, A. Ganner, F. Kotsis, T. Schäfer, H.P.H. Neumann, S. Elsheikh, J. Beck, J.-H. Klingler

**Affiliations:** 1 Department of Neurosurgery, Faculty of Medicine, Medical Center - University of Freiburg, University of Freiburg, Breisacher Str. 64, 79106 Freiburg, Germany; 2 Berta-Ottenstein-Programme for Clinician Scientists, Medical Center - University of Freiburg, Freiburg, Germany; 3 Department of Pediatric Hematology and Oncology, Faculty of Medicine, Medical Center - University of Freiburg, University of Freiburg, Freiburg, Germany; 4 Renal Division, Department of Medicine, Faculty of Medicine, Medical Center - University of Freiburg, University of Freiburg, Freiburg, Germany; 5 Department of Neuroradiology, Faculty of Medicine, Medical Center - University of Freiburg, University of Freiburg, Freiburg, Germany

**Keywords:** Von Hippel-Lindau, Hemangioblastoma, Adolescence, Surveillance, Pediatric neurosurgery

## Abstract

**Purpose:**

Von Hippel-Lindau (VHL) disease is an autosomal-dominantly inherited tumor predisposition syndrome. One of the most common tumors are central nervous system (CNS) hemangioblastomas. Recommendations on the initiation and continuation of the screening and surveillance program for CNS tumors in pediatric VHL patients are based on small case series and thus low evidence level. To derive more robust screening recommendations, we report on the largest monocentric pediatric cohort of VHL patients.

**Methods:**

We performed a retrospective analysis on a pediatric cohort of 99 VHL patients consulted at our VHL center from 1992 to 2023. Clinical, surgical, genetic, and imaging data were collected and statistically analyzed.

**Results:**

42 patients (50% male) developed CNS hemangioblastomas, of whom 18 patients (56% male) underwent hemangioblastoma surgery (mean age at first surgery: 14.9 ± 1.9 years; range 10.2–17). The first asymptomatic patient was operated on at the age of 13.2 years due to tumor progress. Truncating *VHL* mutation carriers had a significantly higher manifestation rate (HR = 3.7, 95% CI: 1.9–7.4, *p* < 0.0001) and surgery rate (HR = 3.3, 95% CI: 1.2–8.9, *p* = 0.02) compared with missense mutation carriers.

**Conclusion:**

We recommend starting MRI imaging at the age of 12 years with examination intervals every (1-) 2 years depending on CNS involvement. Special attention should be paid to patients with truncating variants. Affected families should be educated regularly on potential tumor-associated symptoms to enable timely MRI imaging and eventually intervention, as CNS hemangioblastoma may develop before screening begins.

**German clinical trials Register registration number:**

DRKS00029553, date of registration 08/16/2022, retrospectively registered.

**Supplementary Information:**

The online version contains supplementary material available at 10.1007/s11060-024-04676-5.

## Introduction

Von Hippel-Lindau (VHL) disease is a syndrome of familial predisposition to the development of various malignant and benign tumors caused by inactivation of the *VHL* tumor suppressor gene [1–4]. Central nervous system (CNS) hemangioblastomas are predominantly diagnosed in VHL patients from the third decade of life onwards [1, 3, 5–8].

VHL-associated as well as sporadic hemangioblastomas in children and adolescents are extremely rare (incidence < 1:1,000,000) [9]. The earliest detection was reported in a 6-year-old [10].

Although CNS hemangioblastomas are benign, they can cause significant irreversible neurological deficits depending on their location and lesion size [11, 12]. The growth of hemangioblastomas is unpredictable as they follow multiple growth patterns [11, 13]. Early detection of manifestations through systematic surveillance is considered critical for timely surgical intervention or intensified follow-up MRI scans.

Different screening protocols with varying age of entry and interval of examinations have been proposed to detect or monitor CNS hemangioblastomas in children and adolescents with VHL disease [6, 9, 14–18]. Due to the very low incidence of hemangioblastomas in VHL patients in childhood and adolescence, these recommendations are based on small case series or expert opinions and therefore have a low level of evidence. We conducted a comprehensive study on 99 VHL pediatric patients diagnosed and consulted at our VHL center. Follow up data were available and were analyzed here over more than three decades.

## Materials and methods

### Patient population/study design

The retrospective study cohort consists of VHL patients born between 1976 and 2011 who underwent craniospinal MRI scans between 1992 and 2023 during childhood/adolescence. All patients were analyzed from the age at which they underwent their initial craniospinal MRI examination until (i) the age of 18 (79%) or (ii) the age at time of study inclusion in the case of minors (21%). All patients had confirmed diagnosis of VHL disease based on either genetic (97 patients) or clinical criteria (2 patients) [2]. We collected clinical and imaging data from available patient records and radiographic studies. Patients in which a CNS hemangioblastoma was surgically removed were categorized into two groups based on whether the underlying indication was based on scheduled MRI surveillance (assignment to asymptomatic group) or unscheduled MRI studies because of clinical or neurological symptoms (assignment to symptomatic group). *VHL* germline mutations were classified as either missense (point mutation, microinsertion/-deletion without a frameshift) or truncating (frameshift, nonsense, splice-site mutations, deletions) [19].

### Imaging evaluation

All CNS hemangioblastomas were radiologically assessed in terms of location, size, and mass effect caused by the solid tumor portion, associated cyst and edema formation. The local protocol consists of a high-resolution triplanar contrast-enhanced T1-weighted cranial sequence. The upper and lower spine sequences include sagittal T1-weighted 3-mm thick slices. To accurately assess suspicious lesions in the spinal canal, additional axial 3-mm thick slices were performed. Solid tumor portions and associated cysts were measured in T1-weighted sequences after contrast agent administration. For peritumoral cysts, T2-weighted sequences were also used if available.

Tumor volumes of hemangioblastomas and associated cysts were calculated as the maximum diameter in all three coordinate planes using the ellipsoid formula 0.5 x length x width x height [11, 13, 20]. In cystic tumors, the volume determination was performed separately for the solid and cystic components, as well as for the resulting combined mass effect. Disease progression was determined by the manifestation of a new lesion or the increase in size of an existing lesion greater than 7.5 mm³ per year [11].

### Statistical analysis

Patient characteristics and hemangioblastoma features were summarized using descriptive statistics. The Kaplan-Meier method was employed to estimate the cumulative proportion of patients diagnosed and operated with their first VHL-related CNS hemangioblastoma before the age of 18. The mean was reported with the standard deviation (± SD), a p-value of less than 0.05 was considered statistically significant. Statistical analyses were performed using SPSS software (version 29.0) and GraphPad Prism (version 9.4.1).

## Results

### Patient population

In total, 99 patients (52% male) fulfilled the inclusion criteria, which required genetic or clinical confirmation of VHL disease and at least one craniospinal MRI before the age of 18.

### Organ involvement

The most common manifestation (52%) were retinal angiomatosis. Following retinal involvement, CNS hemangioblastomas were the most frequently observed VHL-associated condition (42%). In descending order of frequency, patients developed pancreatic cysts (37%), pheochromocytomas (20%), renal cysts (9%), pancreatic neuroendocrine tumors (PNET) (5%), and endolymphatic sac tumors (ELST) (3%) before reaching adulthood (Table 1). In 16 patients, no manifestation of the syndrome occurred, with 8 of these patients still being underage at the end of the study, thus no complete observation period was available for these patients.

**Table 1:** Patient characteristics

Variable	Total *n* = 99	Mean ± SD	Median	Range
Female: male	48: 51			
Age at first MRI scan (yrs) Age at first CNS operation (yrs)		13.0 ± 3.0 14.9 ± 1.9	12.7 15.2	1.3–18.0 10.2–17.0
Age at first VHL associated condition		12.7 ± 3.6	13.1	0.0–18.0
Age at first manifestation of:				
**CNS hemangioblastoma** Retinal angiomatosis Phaeochromocytoma Pancreatic cyst Renal cyst PNET ELST	**42** 51 20 37 9 5 3	**14.4 ± 2.5** 13.0 ± 4.0 12.8 ± 3.5 14.5 ± 2.4 14.7 ± 1.7 15.9 ± 1.9 14.5 ± 3.0	**14.8** 13.7 14 14.9 14.8 15.3 13.1	**9.2–18.0** 0.0-17.9 6.8–18.0 8.4–18.0 12.6–17.4 13.4–17.8 12.4–18.0
Mutation				
Missense c.292 T > C Truncating Unknown	60 35 36 3			
Number of CNS HB/patient				
1 2 3 4 5 6–10 > 10	7 10 5 6 5 6 3	4.8 ± 6.3	3	1–39

PNET, pancreatic neuroendocrine tumor; ELST, endolymphatic sac tumor; HB, hemangioblastoma. The surveillance programs were not initiated at a specific age for all patients, as the diagnosis of VHL occurred at different ages

### CNS hemangioblastoma manifestation and surgery

Forty-two patients (50% male) had at least one CNS hemangioblastoma diagnosed before the age of 18 years (mean age 14.4, median age at diagnosis: 14.8 years (range: 9.2–18 years)) (Table 1). Nineteen patients (45% of patients with CNS hemangioblastoma) developed synchronous intracranial (supratentorial, cerebellar, brain stem) and intraspinal lesions. In 22% of patients with CNS hemangioblastoma, only spinal tumors occurred until the age of 18, while in 33% exclusively intracranial tumors were observed. Twenty-five (60%) of the 42 patients with CNS involvement developed multiple CNS hemangioblastomas (≥ 3) by the age of 18 (Table 1).

**Fig. 1:**
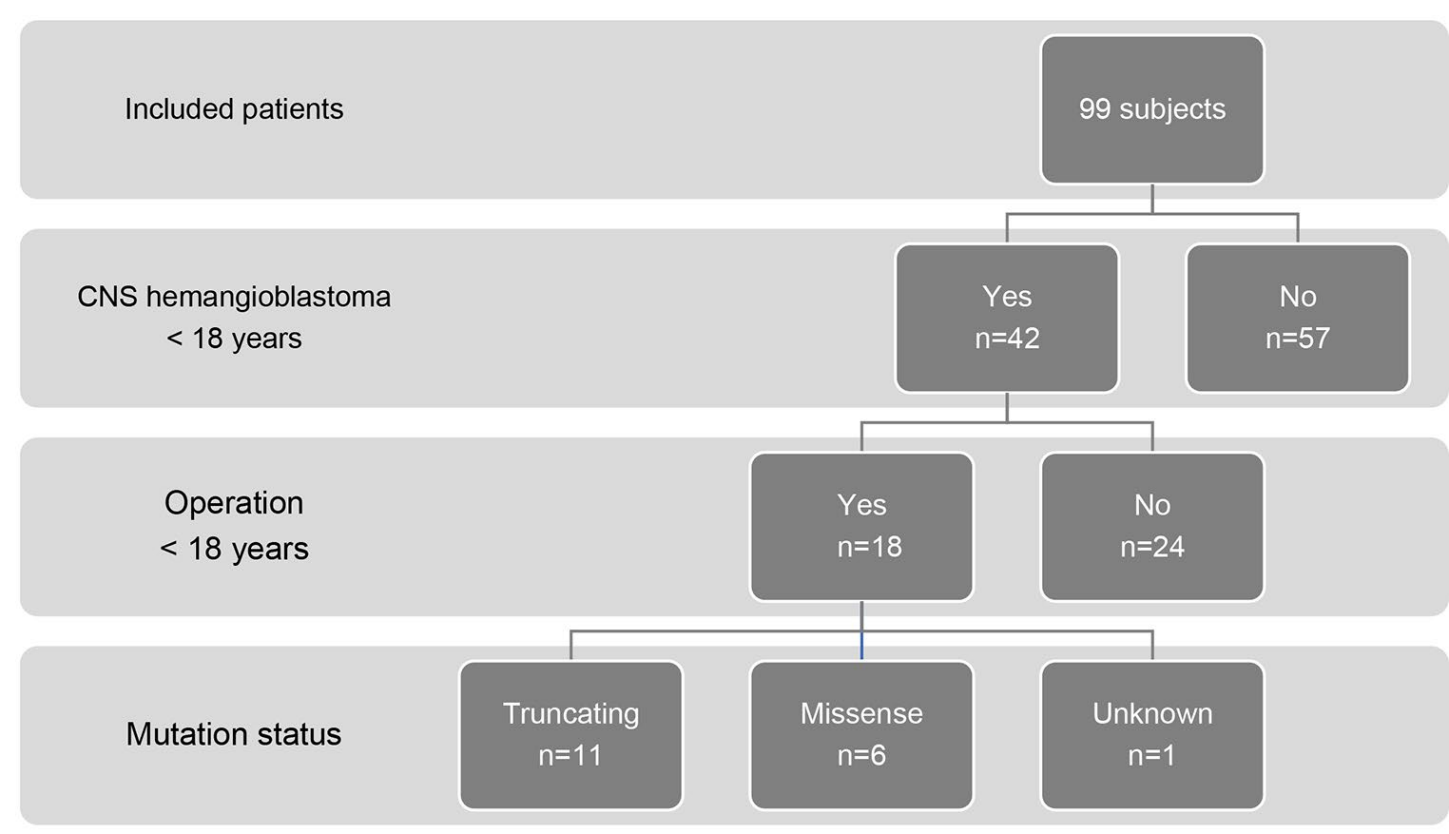
Detection of CNS hemangioblastomas, requirement of surgery and mutation status in the 99 included pediatric VHL patients

Of 42 patients with CNS hemangioblastomas, 18 patients (56% male) underwent hemangioblastoma surgery (Fig. 1).

One patient underwent a total of four surgeries before reaching adulthood, resulting in a total of 21 operations performed on 18 patients. No surgery was required with a likelihood of over 95% before the age of 13. Both patients who underwent surgery before the age of 13 had developed neurological symptoms, leading to the detection of these tumors in the MRI scan.

**Fig. 2:**
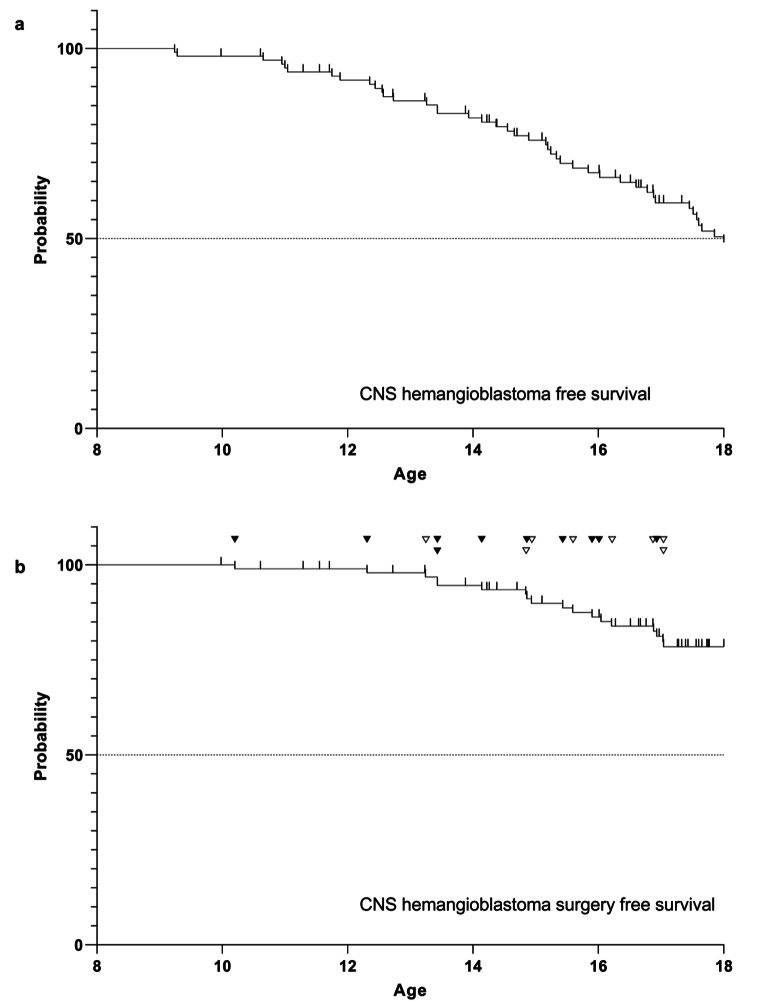
Kaplan-Meier graph showing **A**) the CNS hemangioblastoma free survival and **B**) the CNS hemangioblastoma surgery free survival until the age of 18 years. The indications for surgery made during routine MRI surveillance protocols are shown as white triangles (asymptomatic group), while surgical indication because of MRIs performed in response to symptomatic HB and subsequently leading to a surgical indication are depicted as black triangles (symptomatic group)

The probability to develop hemangioblastoma before reaching adulthood was approximately 50% (median hemangioblastoma free survival 18.0 years) (Fig. 2a).

The first clinically asymptomatic patient with radiologically confirmed tumor growth was operated on at the age of 13.2 years (Fig. 2b).

### Surgical details

All surgeries were performed due to progressive clinical symptoms or signs of mass effect on imaging. Eight patients remained asymptomatic until the time of their first surgery but had radiologically validated tumor volume progression and signs of mass effect on imaging. In 2 operations, up to 3 adjacent hemangioblastomas were resected during the same surgical procedure, resulting in a total of 24 tumors being removed. The surgical procedures were performed on lesions located in the cerebellum (*n* = 11), brain stem (*n* = 9) and spine (*n* = 4). The median operation time was 209 min (208.3 ± 95.4, range 83–367), the median postoperative hospital stay was 7 days (range 4-140).

In one case total resection was unachievable, and in 2 cases, postoperative MRI (partly as late as > 1 year postoperatively) showed minor contrast enhancement in the resection area, so that no clear distinction could be made from subtotal resection, recurrent tumor or inconclusive contrast enhancement. Apart from this, no patient experienced tumor recurrence at the resection site during a mean follow-up period of 17.1 ± 19.8 months (range 0–54).

### Impact of genotype

**Fig. 3:**
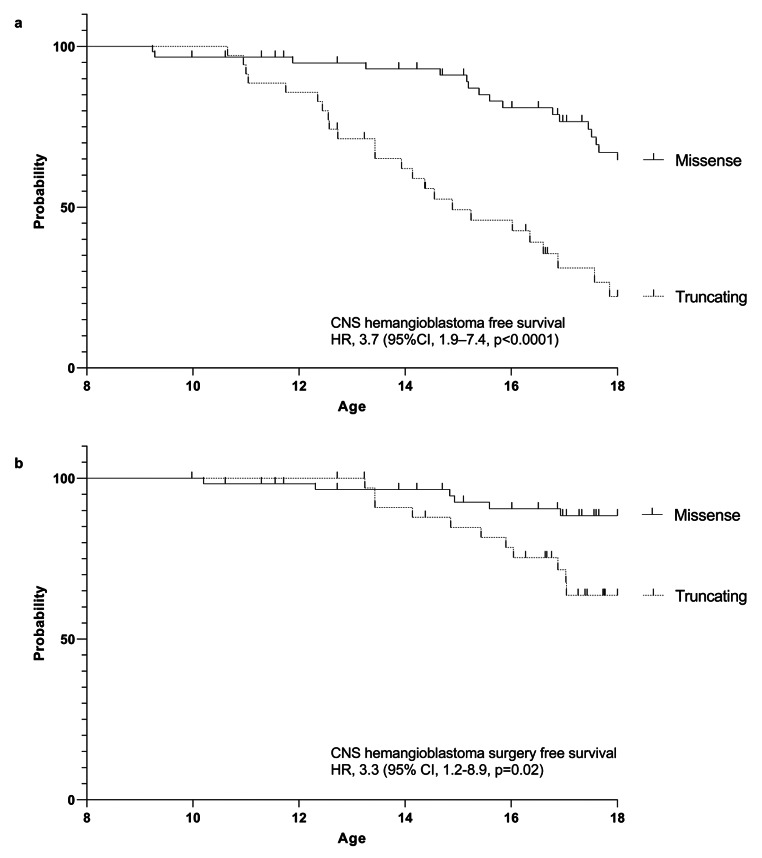
Kaplan -Meier graphs showing (A) the CNS hemangioblastoma free survival (hazard ratio = 3.7, 95% confidence interval: 1.9–7.4, p value < 0.0001) and (B) the CNS hemangioblastoma surgery free survival (hazard ratio = 3.3, 95% confidence interval: 1.2–8.9, p value = 0.02) for carriers of truncating and missense *VHL* mutations

Truncating *VHL* mutations were identified in 36 patients and missense *VHL* mutations were found in 60 patients with a predominance of the “black forest” (c.292T > C; pTyp98His) missense mutation in 35 patients, which is specific to the South German cohort and not mirrored internationally (Table 1). Overall, 35 distinct variant types were identified (Online Resource 1). Truncating *VHL* mutation carriers had a significantly higher manifestation rate compared with missense mutation carriers (hazard ratio = 3.7, 95% confidence interval: 1.9–7.4, p value < 0.0001) (Fig. 3a). Upon reaching adulthood, 72% of individuals carrying missense mutations were unaffected by CNS hemangioblastomas, while for truncating mutation carriers, the percentage was 33%.

Of the 18 operated pediatric VHL patients, six patients were carriers of a missense mutation, 11 patients had a truncating mutation, and the mutation status was unknown for one patient (Fig. 1). Surgery was performed in 10% of patients with missense mutations and in 31% of patients with truncating mutations, leading to a significantly higher rate of CNS hemangioblastoma surgery in truncating *VHL* mutation carriers compared to missense mutation carriers (hazard ratio = 3.3, 95% confidence interval: 1.2–8.9, p value = 0.02) (Fig. 3b). Pediatric patients with missense mutations were operated on at an average age of 14.1 ± 2.4 years (range 10.2–16.9), while for patients with truncating mutations, the average age was 15.2 ± 1.5 years (range 13.2–17.0) (*p* = 0.35). No statistically significant gender-specific difference was found regarding manifestation (*p* = 0.95) or surgery rate (*p* = 0.58).

### Imaging evaluation

The mean age at the first MRI scan was 13.0 ± 3.0 years (range 1.3–18). In non-operated patients, the mean screening interval between CNS imaging was 2.3 ± 1.0 years (range 0.9–5.8), for 27% of the non-operated patients exclusively one MRI scan was available. Among the 42 patients with CNS hemangioblastomas, a total of 201 lesions were detected and analyzed. The mean follow-up period for CNS hemangioblastomas was 2.02 ± 2.03 years (range 0-7.13). The growth behavior of 54 hemangioblastomas (27%) could not be assessed as no additional MRI was performed before the end of the study. Two-thirds (*n* = 98) of the tumors remained stable during the follow-up period, while one-third (*n* = 49) showed progressive growth (defined as > 7.5 mm³/year). Cystic hemangioblastomas exhibited size progression more frequently (*p* < 0.001) and had a significantly larger volume than solid tumors (*p* < 0.001). The follow-up period was less than one year for 42 tumors (21%), one to three years for 45 tumors (22%), and over three years for 60 tumors (30%).

Most CNS hemangioblastomas could be classified as asymptomatic and were identified because of surveillance. More than half of all tumors were found spinal, particularly in the thoracic region. One-third of the hemangioblastomas affected the cerebellum, and 12% were detected in the brainstem. Solid tumors were observed more frequently throughout the neuroaxis than cystic tumors (solid tumors: cerebellum 91%, spinal 90%, and brain stem 67%). Thirteen (54%) of 24 operated hemangioblastomas had tumor associated cysts, while only 13 (7%) of 177 not operated hemangioblastomas had an associated cyst (Table 2).

**Table 2:** Hemangioblastoma characteristics

	All CNS HB *n* = 201 (%)	Operated CNS HB *n* = 24 (%)	Preoperative symptoms (n)
**Supratentorial** solid cystic	2 (1) 1 1	0	
**Cerebellum** solid cystic mixed	65 (32) 59 4 2	11 (46) 5 4 2	headache (4), vertigo (3), diplopia (2), ataxia (2), emesis (2), hydrocephalus (1), singultus (1), asymptomatic (4)
**Brainstem** solid cystic	24 (12) 16 8	9 (38) 3 6	headache (3), emesis (3), ataxia (1), hydrocephalus (1), singultus (1), dysmetria (1), asymptomatic (4)
**Spinal**	110 (55)	4 (17)	
Cervical	34 (17)	1 (4)	
solid	32	1	asymptomatic (1)
cystic	2		
Thoracic	71 (35)	3 (12)	
solid	62	2	
cystic	9	1	
Lumbar	5 (3)	0	sensory deficit (2), pain (1), asymptomatic (1)
solid	5		

Frequency distribution of CNS hemangioblastomas (HB) and symptoms based on anatomical location and type (solid, peritumoral cyst = cystic, intratumoral cyst = mixed)

## Discussion

Due to the overall rarity of VHL disease and the even scarcer occurrence of CNS hemangioblastomas in pediatric patients, existing screening recommendations are mainly based on small case series and expert opinions. With 99 patients, our study comprises the largest single center cohort. The age of initial manifestation of CNS hemangioblastomas in our study (mean age: 14.4, median: 14.8 years, range: 9–18 years) was higher compared to the results of international VHL cohorts (median age: 13–14 years, range: 6–17 years) [10]. At the time of the study, the screening regimen practiced at our VHL reference center suggested a baseline MRI of the CNS at the age of 14 years [9], which may have contributed to this higher age.

In our study retinal hemangioblastomas (52%) and CNS hemangioblastomas (42%) were the most common manifestation in pediatric VHL patients, this is consistent with previous reports [10]. Our reported frequency distribution of hemangioblastomas across anatomical sections of the CNS corresponds to the literature [3, 8, 21].

Recently updated Danish surveillance guidelines have suggested a baseline MRI scan of the CNS at 10 years of age, followed by MRI of the CNS every second year from the age of 15 years [14]. The American VHL Alliance and a recently published consensus statement by the CNS Hemangioblastoma Subcommittee of the International VHL Surveillance Guidelines Consortium recommend shorter screening intervals in adolescents with biennial MRI scans starting at age 11 [6, 15]. Other groups have advocated a baseline MRI as early as 8 years [16] or at even later start ages of 14–15 years [9, 17, 18].

Determining an optimal age for a baseline MRI requires a balance between early tumor detection on the one hand and the necessity of general anesthesia or psychological effects of screening initiation in early childhood on the other. Based on our findings we have revised the previous Freiburg screening protocol starting MRI imaging at the age of 14 years [9] and recommend starting at the age of 12 years. CNS hemangioblastoma may develop earlier in individual cases than in our cohort, and any neurologic symptoms should lead to timely MRI imaging.

Differing CNS hemangioblastoma burden and age-dependent manifestation rate profiles for the two genotype groups have been reported [5, 11, 22, 23]. The large cohort examined in this study confirmed a significantly higher manifestation rate (hazard ratio = 3.7, 95% confidence interval: 1.9–7.4, p value < 0.0001) and surgery rate (hazard ratio = 3.3, 95% confidence interval: 1.2–8.9, p value = 0.02) for truncating compared with missense mutation carriers (Fig. 3). So far, no specific genetically stratified surveillance protocol has been proposed.

We consider it crucial to advise parents affected by VHL to have their children genetically tested at an early stage, as patients with a truncating variant seem to require special attention. In our study, no gender-specific difference was found regarding manifestation and surgery rates, which contrasts with two recently published international VHL cohort studies reporting that tumors grew significantly faster and new tumors developed at a higher frequency in male patients [11, 24].

As previously reported, CNS hemangioblastomas can follow unpredictable growth pattern influenced by age, sex, genotype, associated cysts, and anatomic location [5, 7, 8, 11, 13, 24]. Overall, 50% of CNS hemangioblastomas show no significant change in size over a long-term follow-up period [11]. In our study, 67% of the tumors demonstrated size stability, which could potentially be attributed to the relatively short average follow-up period of 2 years. Symptom-producing hemangioblastomas are frequently associated with cysts, with the growth rate of cysts typically exceeding that of the solid component [25]. We confirmed that hemangioblastomas with cysts were more likely to require surgery as a result of increased lesion volume. The most frequent preoperative symptoms were related to increased intracranial pressure or cerebellar symptoms such as headache, vertigo, diplopia, emesis, and ataxia (Table 2) which is consistent with data reported in pediatric CNS hemangioblastomas [26].

Symptomatic tumors are universally agreed to require neurosurgical intervention, while the clinical management of asymptomatic but radiologically progressive tumors varies in the literature. The preoperative neurological status is considered an important factor associated with long-term postoperative outcomes in pediatric patients [21]. To prevent the development of irreversible neurological deficits in pediatric patients, the surgical removal of asymptomatic hemangioblastomas with documented radiological progression has been advocated [27]. Our findings support this recommendation, as surgical procedures for asymptomatic tumors could be performed with low morbidity. Two previous studies have reported favorable clinical outcomes for pediatric CNS hemangioblastoma surgery and no cases of local tumor recurrence [26, 27]. This aligns with our observations, however local tumor recurrence could not be ruled out in two cases.

Adolescent patients require special attention as patients 12 to 20 years of age develop more tumors per year than older age groups and cysts grow faster in younger patients [11, 28]. A personalized surveillance plan should be developed considering hemangioblastoma burden, tumor location, tumor size and associated cysts. We recommend MRI examination intervals every (1-) 2 years depending on CNS involvement. Of 29 patients who underwent follow-up MRI examinations for at least one year (mean follow-up 3.8 ± 1.7, range 1.0-7.1 years) after the initial diagnosis of a CNS hemangioblastoma, 22 patients (76%) showed disease progression defined as the manifestation of a new hemangioblastoma (66%) or growth of an existing lesion > 7.5 mm³/year (59%). The mean time until disease progression occurred was 2.0 ± 1.3 years (range 0.3–5.8). The risk of intercurrent CNS hemangioblastoma was reported to be reduced from 7 to 3% when CNS imaging was performed annually instead of every 2 years [29]. Close observation with annually screening intervals may reduce neurological morbidity but must be balanced against potential psychological effects of frequent screening intervals and risks associated with general anesthesia or contrast agent accumulation. For asymptomatic patients with stable or no hemangioblastomas we consider a biennial interval for MRI scans reasonable. Patients with a high tumor burden or progressive hemangioblastoma, especially when associated with cysts, require more frequent screening intervals to weight between watchful waiting and intervention. If new neurological symptoms emerge, an MRI should be scheduled to guide treatment. Patients and even more their parents should be educated on possible cerebellar or spinal symptoms and signs of increased intracranial pressure to raise clinical awareness and enable early intervention. To reduce the psychological burden of frequent examinations and to increase compliance, we recommend a multidisciplinary “one-stop-shop” service for clinical appointments so that all organs potentially affected by VHL disease are examined in one day [30].

### Limitations

As retrospective investigation, this study includes VHL patients followed in our VHL center for whom MRI examinations and surgeries were collected over longer periods of time, so that complete clinical information was not always available. The burden of tumor lesions at the age of the first craniospinal MRI examination depends on the scheduling of the first screening examination, so that it is hardly possible to make a statement about the earlier dynamics of tumor growth. Since the end of observation was reaching adulthood, the follow-up period for the individual lesions was too limited to conduct a comprehensive analysis of tumor growth patterns stratified on anatomic locations. The predominance of the c. 292 T > C mutation among the missense mutation carriers in our cohort may limit the international applicability of our findings. Nevertheless, this is the largest pediatric VHL patient cohort evaluating recommended initiation of routine MRI monitoring of the CNS.

## Conclusion

We investigated the largest single-center cohort of pediatric VHL patients to derive a protocol for the initiation of CNS hemangioblastoma surveillance and the appropriate intervals for its continuation. Our recommendation is starting MRI surveillance at the age of 12 years with regular examination intervals every (1-) 2 years depending on CNS involvement. Children of patients affected by VHL should be genetically tested at an early age as patients with truncating variant are associated with a nearly four times higher risk of hemangioblastoma development and threefold increased risk for surgical intervention compared to patients with missense variant. Affected families should be educated on potential neurological symptoms and signs of increased intracranial pressure to allow surgical removal before irreversible neurological deficits occur.

### Electronic supplementary material

Below is the link to the electronic supplementary material.

Supplementary Material 1

## Data Availability

Data will be made available upon reasonable request.
