# Supplementary material for: Screening and surveillance recommendations for central nervous system hemangioblastomas in pediatric patients with Von Hippel-Lindau disease
Source: J Neurooncol. 2024 Apr 22;168(3):537–45. doi: 10.1007/s11060-024-04676-5 (PMC11186940; doi:10.1007/s11060-024-04676-5)
Supplement: Supplementary file 1 — Supplementary Material 1 [file 11060_2024_4676_MOESM1_ESM.pdf]

Supplementary Material

Journal of Neuro-Oncology

Screening and surveillance recommendations for central nervous system hemangioblastomas in pediatric patients with von Hippel-Lindau disease

A.L. Knoblauch[1], B.-I. Blaß[1], C. Steiert[1], N. Neidert[1,2], A. Puzik[3], E. Neumann-Haefelin[4], A. Ganner[4], F. Kotsis[4], T. Schäfer[4], H.P.H. Neumann[4], S. Elsheikh[5], J. Beck[1], J.-H. Klingler[1]

[1] Department of Neurosurgery, Medical Center - University of Freiburg, Faculty of Medicine, University of Freiburg, Breisacher Str. 64, 79106 Freiburg, Germany

[2] Berta-Ottenstein-Programme for Clinician Scientists Medical Center, University of Freiburg, Freiburg, Germany

[3] Department of Pediatric Hematology and Oncology, Medical Center - University of Freiburg, Faculty of Medicine, University of Freiburg, Freiburg, Germany

[4] Renal Division, Department of Medicine, Medical Center - University of Freiburg, Faculty of Medicine, University of Freiburg, Freiburg, Germany

[5] Department of Neuroradiology, Medical Center - University of Freiburg, Faculty of Medicine, University of Freiburg, Freiburg, Germany

Corresponding Author: Anna Laura Knoblauch. Department of Neurosurgery, Medical Center - University of Freiburg, Breisacher Str. 64, D-79106 Freiburg, Germany, annalaura.knoblauch@web.de

Supplement Table 1: Germline *VHL* mutations

Exon	Nucleotide change new nomenclature	Protein change	Mutation type	No. of patients w/ HB[a]	No. of operations[b]
1	93 G>A	Glu31Asp	Missense	0/2	
1	194 C>A	Ser65stop	Nonsense	2/2	1/2
1	208 G>T	Glu70stop	Nonsense	1/1	1/1
1	221 T>G	Val74Gly	Missense	1/1	1/1
1	224_226 del TCT o. 227_230 del TCT	76delF	In frame deletion	1/1	1/1
1	235 C>G	Arg79Gly	Missense	1/1	0/1
1	236_241 del	R79S 80 del	In frame deletion	1/1	0/1
1	239 G>T	Ser80Ile	Missense	1/2	0/2
1	239_240insC	Pro81Ser*fs51	Frameshift	1/1	1/1
1	240 T>G	Ser80Arg	Missense	1/1	1/1
1	241 C>T	Pro81Ser	Missense	0/1	
1	266 T>C	Leu89Pro	Missense	0/3	
1	289 ins 17 bp		Frameshift	0/1	
1	292 T>C	Tyr98His	Missense	4/35	1/35
1	319 C>G	Arg107Gly	Missense	1/1	1/1
1	320 G>A	Arg107His	Missense	0/1	
2	397 A>C	Thr133Pro	Missense	1/1	0/1
2	445_458 del	N150S	Frameshift	0/1	
2	461 C>T	Pro154Leu	Missense	1/1	0/1
3	464-2 A>G		Splice	1/1	1/1
3	467 A>G	Tyr156Cys	Missense	0/1	
3	472 C>G	Leu158Val	Missense	1/1	0/1
3	481 C>T	Arg161stop	Nonsense	1/4	1/4
3	482 G>A	Arg161Gln	Missense	0/1	
3	486 C>G	Cys162Trp	Missense	1/1	1/1
3	490 C>T	Gln164stop	Nonsense	1/2	0/2
3	500 G>A	Arg 167Gln	Missense	1/1	0/1
3	533 T>A	Leu178Gln	Missense	1/1	0/1
3	548 C>A	Ser183stop	Nonsense	1/1	1/1
3	593 T>A	Leu198Gln	Missense	0/2	
	Deletion Exon 1		Deletion	0/1	
	Deletion Exon 2		Deletion	2/2	0/2
	Deletion Exon 3		Deletion	4/4	3/4
	Deletion Exon 1,2		Deletion	1/1	0/1
	Deletion Exon 1-3		Deletion	9/14	2/14
	unknown			1/3	1/3

Overview of the *VHL* disease germline mutations found in our patient population. [a]Number of patients with CNS hemangioblastomas/all patients with this specific nucleotide change of *VHL* germline mutation and [b]number of operated patients for CNS hemangioblastoma/all patients with this specific nucleotide change of *VHL* germline mutation.
